# Investigating the Link Between Low Back Ache, Abdominal Discomfort and Oliguria: A Unique Case of Histopathology-Proven Retroperitoneal Fibrosis

**DOI:** 10.7759/cureus.44220

**Published:** 2023-08-27

**Authors:** Jithin Vijayan, Hrishikesh Bora, Amir Ali, Devika K R

**Affiliations:** 1 Medicine, Downtown Hospital, Guwahati, IND; 2 Pharmacy, National Institute of Pharmaceutical Education and Research, Guwahati, IND; 3 Radiodiagnosis, Fakhruddin Ali Ahmed Medical College and Hospital, Barpeta, IND

**Keywords:** immunosuppressants, corticosteroids, histopathology, biopsy, back ache, abdominal pain, inflammation, retroperitoneal fibrosis

## Abstract

Retroperitoneal fibrosis (RPF) is a rare condition characterized by systemic inflammation and the proliferation of fibroinflammatory tissues in the retroperitoneum. It may lead to the formation of a retroperitoneal mass and can encase the aorta, its branches and ureters. The pathogenesis of RPF is not fully known. We report a case of a 52-year-old male presented with low back ache, flank pain, swelling of legs, oliguria and features of obstructive uropathy, later diagnosed to be RPF. The mainstay of diagnosis includes blood workup, imaging and biopsy. The first line of treatment is corticosteroids. Surgical intervention is carried out when medical measures have failed or when contraindicated. Early diagnosis and prevention of complications is the key, and a high degree of suspicion is needed.

## Introduction

Retroperitoneal fibrosis is a chronic inflammation of fibrosclerotic tissues of the retroperitoneum which affects the aorta, its branches and ureters. It is uncommon with an estimated incidence of 1.38 cases per one lakh people [1]. It is characterized by flank pain, dull or poorly localized abdominal pain and symptoms such as weight loss, anorexia, fever and malaise [2]. Other symptoms include ureteric colic, haematuria, lower limb oedema and constipation which are associated with malignancies, infections, and conditions like hypertension and dyslipidemia. Computed tomography (CT) scans and magnetic resonance imaging (MRI) can be helpful [3]. Corticosteroids (CS) remain the therapy of choice, and other immunomodulators are used. Despite the high success rates, relapses after cessation of CS are recorded in 24% to 72% of patients [4]. Surgery is preferred only after conservative management fails or is contraindicated.

Retroperitoneal fibrosis (RPF) is relatively rare, there are limited randomized controlled trials and there are no universal guidelines for treatment. Comparing different treatment strategies and biopsy of the fibrosclerotic tissues remains the mainstay. Here, we report a rare case of RPF confirmed by histopathology.

## Case presentation

A 52-year-old male patient came to the hospital with a history of low back ache and flank pain for two to three months; reduced urine output, frequency and abdominal discomfort for four to five days; and bilateral swelling of legs for four to five days. Back ache was chronic, insidious in onset and aching type. It was aggravated by doing heavy work. No history of dysuria or haematuria, nausea, vomiting, generalized swelling, shortness of breath, chest pain, orthopnoea or paroxysmal nocturnal dyspnoea was present. He is not a known case of hypertension/type 2 diabetes/coronary artery disease or other systemic illness.

**Figure 1:**
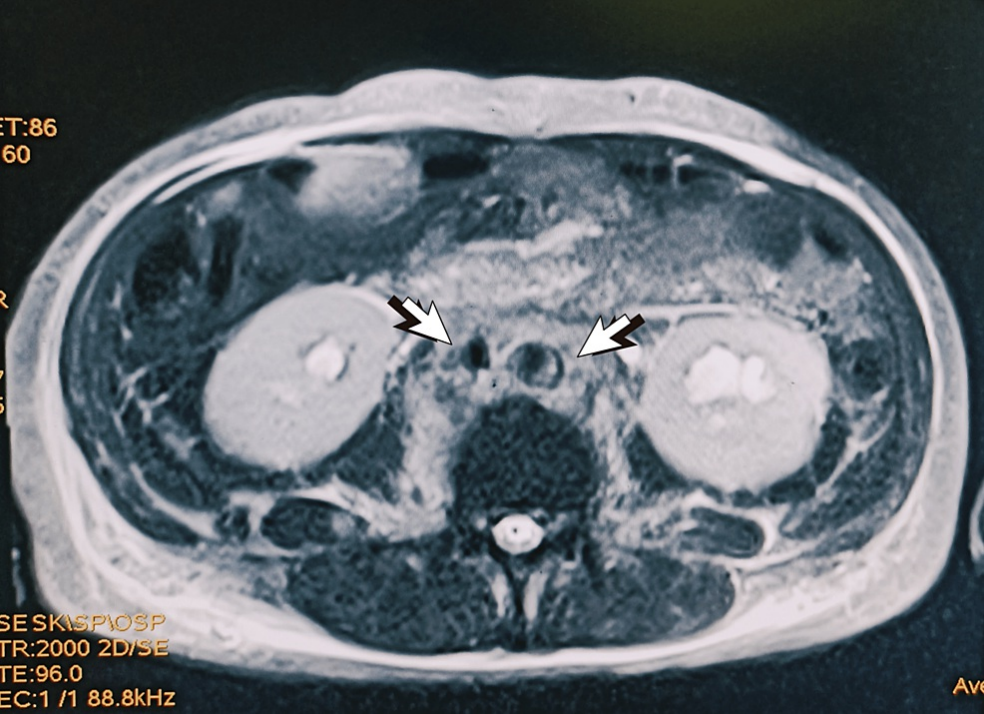
Axial T2W image of the abdomen showing heterogenous soft tissue encasing IVC and abdominal aorta (white arrows) T2W: T2-weighted image, IVC: inferior vena cava.

**Figure 2:**
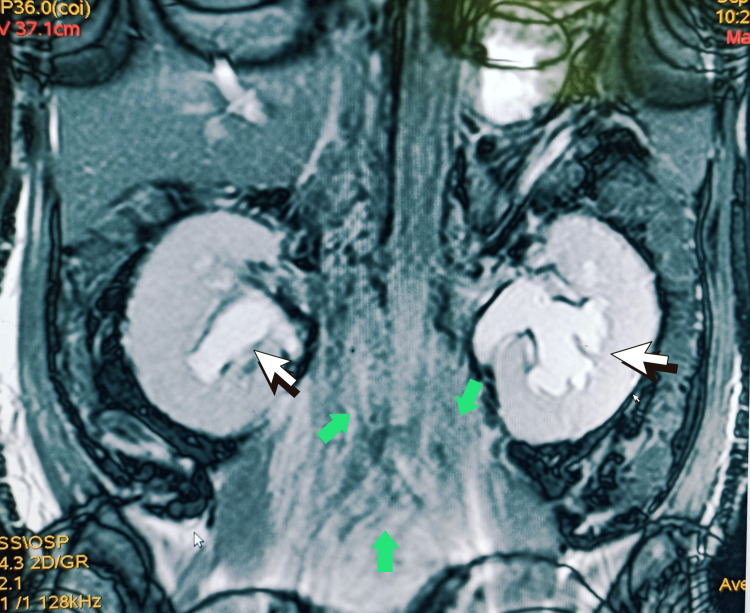
T2W image showing heterogenous soft tissue encasing abdominal aorta extending up to bifurcation (green arrows) Mild fullness of the pelvicalyceal system of bilateral kidneys (white arrows). T2W: T2-weighted image.

**Figure 3:**
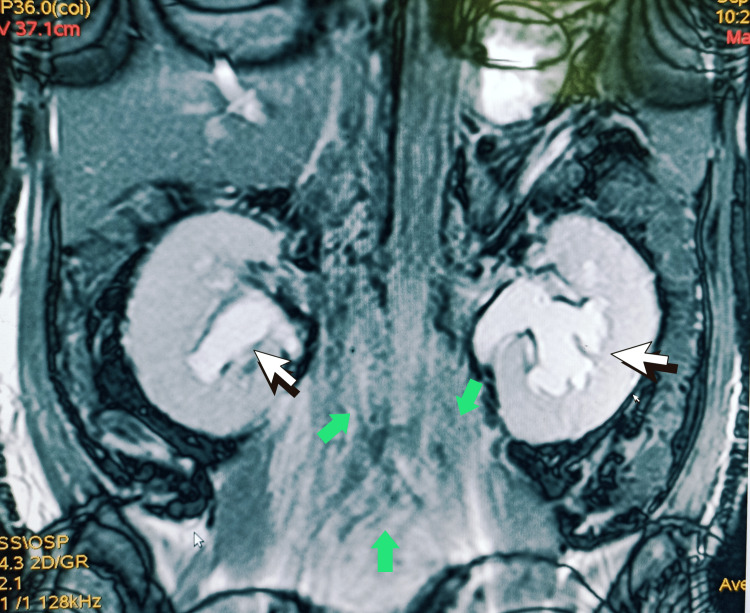
T2W image showing heterogenous soft tissue encasing abdominal aorta extending up to bifurcation (green arrows) Mild fullness of the pelvicalyceal system of bilateral kidneys suggestive of hydroureteronephrosis due to ureteric obstruction by the retroperitoneal soft tissue (white arrows). T2W: T2-weighted image.

The patient was conscious, oriented and alert and moderately built and nourished. On physical examination, pallor and bilateral pedal oedema were noted. On examination, he was afebrile, his pulse rate was 70 beats per minute and elevated blood pressure (160/100 mm of Hg) was noted. Respiratory and cardiovascular examinations were normal. The abdomen was slightly distended, with no superficial lymph nodes, no organomegaly and no renal angle tenderness. On laboratory investigations, haemoglobin was 10 g/dl; total white blood cell count was 12,300/mm^3^; erythrocyte sedimentation rate (ESR) was 35 per hour; and severely elevated serum creatinine levels (14.6 mg/dl), dyselectrolytemia and sodium (Na) 127 mEq per litre were noted. The urine routine showed microscopic haematuria and some epithelial cells. In ultrasonography (USG) of the abdomen, bilateral moderate hydroureteronephrosis with dilated upper ureter was seen. CT abdomen was suggestive of mild heterogeneously enhancing soft tissue component in the retroperitoneum encasing the lower abdominal aorta (infrarenal aorta), iliac vessels and ureters with mild fullness of pelvicalyceal system of left kidney suggestive of RPF. MRI findings were also suggestive of RPF (Figures 1-3).

**Figure 4:**
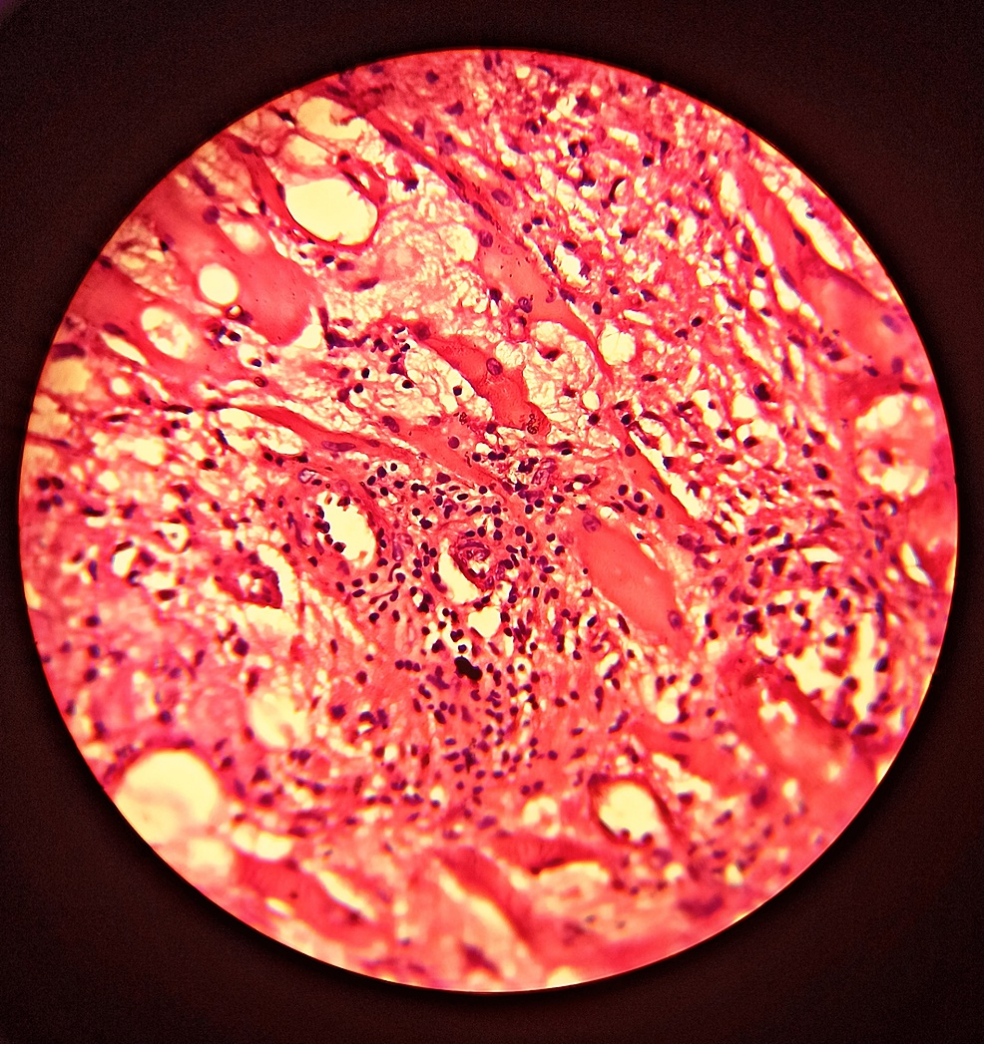
Histology (H&E stain) of the retroperitoneal fibrous tissue High-power microscopy shows fibrous and fatty tissue infiltrated with a group of lymphocytes suggestive of a chronic inflammatory lesion with fibrosis. H&E: haematoxylin and eosin.

**Figure 5:**
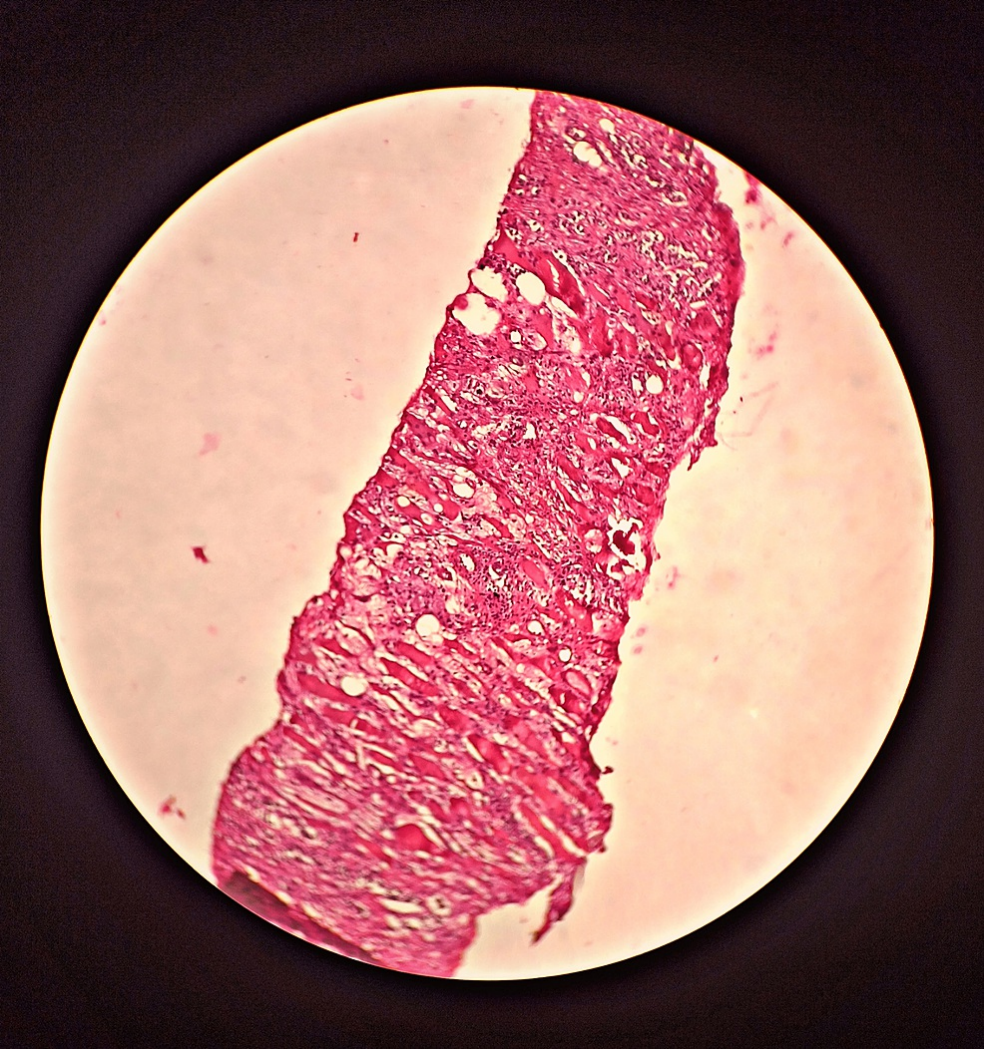
Histology (H&E stain) of the retroperitoneal fibrous tissue in low power Pathology demonstrated fibrous tissue with fatty tissues. H&E: haematoxylin and eosin.

The confirmatory diagnosis was made with a CT-guided biopsy of retroperitoneal tissue which showed elongated strips of tissue composed of loose fibrous tissues, fatty tissues and skeletal muscle fibres suggestive of the chronic inflammatory lesion with fibrosis (Figures 4, 5).

Subsequent reports of Interferon Gamma Release Assay (IGRA) for tuberculosis (TB) and immunoglobulin G4 (IGG4) antibodies were negative. Based on CT, MRI and other investigations, secondary causes like tuberculosis, autoimmune diseases and others were ruled out. Hence, a diagnosis of idiopathic retroperitoneal fibrosis was established.

## Discussion

Retroperitoneal fibrosis is an uncommon disease, and aetiopathogenesis is still unknown. Men are two to three times more likely to be affected than women, and the average age of presentation is 50 to 60 years. Its origin is idiopathic in about 2/3 of the cases, while the remaining 1/3 arise secondary to a number of factors like malignancies, infections, drugs, surgery, injuries and medications (methysergide, ergot derivatives and beta-blockers) [5]. RPF can be caused by autoimmune diseases and IGG4 antibody-related disorders. The clinical manifestations of RPF are generally nonspecific and heterogeneous, making diagnosing this rare condition even more challenging [6]. Classically, there will be a retroperitoneal plaque encasing great vessels and ureters from the renal hilum to the pelvis [7].

Malignancy should be also excluded. In our case, the patient came with dull flank pain, lower limb swelling, fatigue and features of obstructive uropathy. But precipitating factors including drugs were unknown. The preferred diagnostic modalities for RPF are cross-sectional imaging methods like computed tomography (CT) and magnetic resonance imaging (MRI). Retroperitoneal tissue biopsy is recommended to confirm the diagnosis in cases with uncommon presentation and the suspicion of underlying malignancy [3].

Corticosteroids are considered the main treatment stay regardless of the aetiology. Corticosteroids as a monotherapy are the commonly followed regimen, and in severe cases, it can be combined with other immunosuppressants [8,9]. This adds to therapy and aids in minimizing the risks and long-term side effects of steroids [10-12]. An oral steroid (prednisolone) was started along with supportive medications. Renal replacement therapy was essential in view of the severity, and haemodialysis was preferred to relieve kidney functions. The patient needed double J (DJ) stenting to relieve the obstruction. The patient started to improve owing to the concurrent use of corticosteroids, haemodialysis and double J (DJ) stenting.

## Conclusions

Symptoms and signs of RPF are usually vague and non-specific; hence, a high index of suspicion is needed especially for patients with bilateral ureteric obstruction and presenting with features of obstructive uropathy. Any soft tissue mass with obstruction should be investigated properly, and causes need to be evaluated. Autoimmune diseases and tuberculosis need exclusion with relevant investigations. Corticosteroids are the mainstay of therapy, and surgical intervention may be needed to relieve obstructive uropathy and preserve renal function.
